# SMS based external quality assessment of reading and interpretation of malaria rapid diagnostic tests: Preliminary results among more than 2000 end-users in the Democratic Republic of the Congo

**DOI:** 10.1186/1475-2875-11-S1-P65

**Published:** 2012-10-15

**Authors:** Pierre Mukadi, Veerle Leion, Albert Lukuka, Joêl Mbatshi, John Otshudiema, Jean-Jacques Muyembe, Philippe Gillet, Jan Jacobs

**Affiliations:** 1 Institut National de Recherche Biomédical, Kinshasa, Democratic Republic of the Congo; 2 Department of Clinical Sciences, Institute of Tropical Medicine, Antwerp, Belgium; 3 Programme National de Lutte contre le Paludisme, Kinshasa, Democratic Republic of the Congo; 4 Programme National de Lutte centre la Tuberculose, Kinshasa, Democratic Republic of the Congo; 5 Programme Santé Intégré, Kinshasa, Democratic Republic of the Congo; 6 Université de Kinshasa, Kinshasa, Democratic Republic of the Congo

## Background

Rapid diagnostic tests (RDT) are increasingly replacing microscopy for diagnosis of malaria in endemic settings. Although RDTs are simple and robust, errors in the post analytical phase i.e. in reading and interpretation of the RDT result, are not uncommon. In the Democratic Republic of the Congo (DRC) malaria is endemic, and malaria RDTs have been introduced since 2010. In June-July 2012, an external quality assessment (EQA) addressing correct reading and interpretation of the three band malaria RDT recommended by the National Malaria Control Programme was organized among end-users in DRC.

## Materials and methods

High resolution photographs of 10 patient RDT results representing a variety of malaria diagnosis, (combinations of) species, invalid or unreadable tests, faint positive test lines etc. were prepared. Answers were encoded in multiple choice format. Photographs were sent to focal points in 9 out of 11 provinces in DRC, who distributed them to malaria RDTs end-users. For each health facility, 1 questionnaire on availability, training and use of RDTs in the structure accompanied the photographs. End-users were requested to answer the multiple choice individually, by sending a short text message (SMS) to the study coordinator in Kinshasa who transferred SMS by blue tooth to an excel database. Questionnaires were recollected from the health facility by the focal points and sent back to Kinshasa.

## Results

Preliminary results are presented. In total, more than 2000 end-users participated in this EQA. Overall, about one out of 5 participants red and interpreted all 10 photographs correctly. Less than 1% had all 10 answers incorrect. For each individual photograph, between 50-90% of correct answers were received. In up to 40% of answers, the result represented a major error such as not recognizing an invalid test result, not recognizing negative test results or not recognizing a *Plasmodium falciparum* infection. Failure to detect faint or weak test lines was a common reason for wrong answers.

## Conclusions

The current EQA consisted of an innovative combination of a photograph-based approach, as being used in HIV and malaria RDT trainings, with communication by standard cell phone and SMS. Using this approach, we confirmed that errors in reading and interpretation of malaria RDTs are widespread in DRC. Data generated through this study can be exploited by control programs to identify common problems in job aids or end-user’s performance and to organize appropriate remediation. Organisation of regular external quality assessments with feed-back to the participants might as well improve end user performance by its educational stimulus. In addition, recording of basic professional data through EQA, allows future access to the end-users by cell-phone network for malaria RDT-related issues.

